# Evaluating the Leucine Trigger Hypothesis to Explain the Post-prandial Regulation of Muscle Protein Synthesis in Young and Older Adults: A Systematic Review

**DOI:** 10.3389/fnut.2021.685165

**Published:** 2021-07-08

**Authors:** Gabriele Zaromskyte, Konstantinos Prokopidis, Theofilos Ioannidis, Kevin D. Tipton, Oliver C. Witard

**Affiliations:** ^1^Department of Nutritional Sciences, King's College London, London, United Kingdom; ^2^Department of Musculoskeletal Biology, Institute of Life Course and Medical Sciences, University of Liverpool, Liverpool, United Kingdom; ^3^Institute of Performance Nutrition, London, United Kingdom; ^4^Centre for Human and Applied Physiological Sciences, Faculty of Life Sciences and Medicine, King's College London, London, United Kingdom

**Keywords:** blood leucine kinetics, leucine threshold, intact proteins, protein-rich whole foods, skeletal muscle, aging, exercise, muscle hypertrophy

## Abstract

**Background:** The “leucine trigger” hypothesis was originally conceived to explain the post-prandial regulation of muscle protein synthesis (MPS). This hypothesis implicates the magnitude (amplitude and rate) of post-prandial increase in blood leucine concentrations for regulation of the magnitude of MPS response to an ingested protein source. Recent evidence from experimental studies has challenged this theory, with reports of a disconnect between blood leucine concentration profiles and post-prandial rates of MPS in response to protein ingestion.

**Aim:** The primary aim of this systematic review was to qualitatively evaluate the leucine trigger hypothesis to explain the post-prandial regulation of MPS in response to ingested protein at rest and post-exercise in young and older adults. We hypothesized that experimental support for the leucine trigger hypothesis will depend on age, exercise status (rest vs. post-exercise), and type of ingested protein (i.e., isolated proteins vs. protein-rich whole food sources).

**Methods:** This qualitative systematic review extracted data from studies that combined measurements of post-prandial blood leucine concentrations and rates of MPS following ingested protein at rest and following exercise in young and older adults. Data relating to blood leucine concentration profiles and post-prandial MPS rates were extracted from all studies, and reported as providing sufficient or insufficient evidence for the leucine trigger hypothesis.

**Results:** Overall, 16 of the 29 eligible studies provided sufficient evidence to support the leucine trigger hypothesis for explaining divergent post-prandial rates of MPS in response to different ingested protein sources. Of these 16 studies, 13 were conducted in older adults (eight of which conducted measurements post-exercise) and 14 studies included the administration of isolated proteins.

**Conclusion:** This systematic review underscores the merits of the leucine trigger hypothesis for the explanation of the regulation of MPS. However, our data indicate that the leucine trigger hypothesis confers most application in regulating the post-prandial response of MPS to ingested proteins in older adults. Consistent with our hypothesis, we provide data to support the idea that the leucine trigger hypothesis is more relevant within the context of ingesting isolated protein sources rather than protein-rich whole foods. Future mechanistic studies are warranted to understand the complex series of modulatory factors beyond blood leucine concentration profiles within a food matrix that regulate post-prandial rates of MPS.

## Introduction

Dietary protein is widely regarded as crucial for skeletal muscle health and performance across the lifespan. While muscle hypertrophy is a common goal and pre-requisite to success for strength/power-based athletes and exercise enthusiasts, the maintenance of muscle mass and quality also provides a fundamental hallmark of healthy aging. At the metabolic level, muscle mass and quality are dependent on the continuous remodeling of skeletal muscle proteins via temporal fluctuations in rates of muscle protein synthesis (MPS) and muscle protein breakdown (MPB) (1). Over time, the relationship between rates of MPS and MPB dictate the net gain or loss of skeletal muscle protein. Both MPS and MPB are responsive to diet, specifically protein feeding and the subsequent aminoacidemia (2), and mechanical loading including resistance (3), endurance (4), and concurrent (5) exercise modalities. However, of these two metabolic processes, the fold change in MPS with protein feeding or exercise is 4–5 times greater than MPB (6), meaning that MPS is the primary locus of control for muscle protein mass, at least in healthy individuals. Accordingly, understanding the regulation of MPS with protein/amino acid feeding and exercise is fundamental to optimizing protein nutrition recommendations for muscle health and performance, both from athletic and clinical perspectives.

The magnitude of the muscle protein synthetic response to an ingested protein source is regulated on multiple levels of physiology that include, but may not be limited to, (i) the systemic availability of amino acids, (ii) the transport and uptake of amino acids into skeletal muscle, and (iii) the activity of intramuscular cell signaling proteins known to modulate MPS (7). Accordingly, it has been proposed that the anabolic potential of a protein source is dependent on factors related to protein digestibility and amino acid kinetics, and amino acid composition. A longstanding debate within the field of muscle protein metabolism relates to whether MPS is regulated by changes in the intracellular (8) or extracellular (9) availability of amino acids. Mechanistic studies support the notion that a more rapid appearance of dietary protein derived amino acids (10), specifically the essential amino acids (EAA) (11), into the circulation is stimulatory for MPS during post-exercise recovery, albeit not under resting conditions (12). Moreover, of all EAA, the branched-chain amino acid, leucine, has been shown to independently upregulate the muscle protein synthetic machinery by activating the mechanistic target of rapamycin complex 1 (mTORC1) which is an intracellular signaling cascade that switches on the translation initiation process of MPS (13, 14). As a result, the “leucine trigger” hypothesis has been proposed. This hypothesis predicts that the magnitude (amplitude and rate) of post-prandial increase in blood leucine concentrations, termed leucinemia, serves to regulate the magnitude of post-prandial MPS response to an ingested protein source (15–17).

Experimental support for the leucine trigger hypothesis primarily stems from studies of isolated protein sources such as intact whey, micellar casein, and soy protein fractions (15, 16). In this regard, the amplitude of peak post-prandial leucinemia was highest for whey, intermediate for soy and lowest for casein. This hierarchy corresponded to the differential post-prandial response of MPS to each protein source at rest and during exercise recovery (15). Accordingly, this relationship was used as the basis to develop the leucine trigger hypothesis (18). Interestingly, the leucine trigger hypothesis has recently been challenged following observations from a series of experimental studies that revealed an apparent disconnect between blood leucine concentration profiles (i.e., the amplitude and rate of leucinemia) and the MPS response to ingested protein in both young and older adults (19, 20). Moreover, recent studies reported that protein-rich whole food sources also are potent in stimulating MPS, despite not facilitating a rapid rise in leucine concentrations during exercise recovery (19). Therefore, the primary aim of this qualitative systematic review was to examine the influence of blood leucine concentration profiles on the post-prandial regulation of MPS in response to protein ingestion at rest and post-exercise in young and older adults. We hypothesize that experimental support for the leucine trigger hypothesis will depend on several factors, including (i) the demographic characteristics of participants (i.e., age), (ii) exercise status (i.e., rest vs. exercise recovery), and (iii) the dose and source of ingested protein (i.e., isolated proteins vs. protein-rich whole food sources).

## Methods

The methodology for this systematic review is based on the PRISMA 2009 guidelines and a PICOS framework was used to determine the search strategy and study characteristics. Consistent with Shad et al. (21), we chose to qualitatively synthesize data from included studies given the heterogenous methodology used to measure MPS between laboratories, meaning that quantitative analysis across studies was not feasible.

### Search Strategy

A systematic literature search was conducted in PubMed, Scopus, Cochrane, Google Scholar databases, with the final literature search completed on 1st February 2021. These databases were selected to capture the wide range of content in the field of protein nutrition and muscle protein metabolism. A MeSH (Medical Subject Headings) tree method was used to determine the following search terms: (Healthy old adults OR healthy elderly OR older OR elderly OR healthy young adults OR young adults) AND (rest OR exercise OR resistance exercise OR endurance exercise) AND (protein feeding OR protein digestion kinetics OR amino acid ingestion OR protein supplementation OR whey protein OR soy protein OR casein protein OR wheat protein OR milk protein OR whey OR casein OR soy OR wheat OR milk OR leucine OR leucine trigger) AND (protein turnover OR MPS OR muscle protein synthesis OR FSR OR fractional synthetic rate OR protein synthesis OR myofibrillar protein synthesis OR plasma amino acid concentrations OR plasma leucine concentrations OR dietary protein OR protein-rich). Further studies were identified through the reference lists of relevant original articles and review articles.

### Eligibility Criteria

#### Types of Studies

Randomized controlled trials (RCT), non-randomized clinical trials and comparative studies that combined measurements of blood (plasma) leucine concentrations and post-prandial rates of MPS in response to the oral ingestion of two or more different sources of isolated intact proteins or protein-rich whole foods were eligible for inclusion. Only original manuscripts (not abstracts or reviews) written in English were selected and no limitations on publication date were applied.

#### Types of Participants

All studies included in this systematic review were conducted in accordance with ethical standards. Studies that recruited healthy young, middle-aged, or older males or females were included in this systematic review. Young adults were defined in the range of 18–35 y, middle-aged in the range of 35–60 y and older adults in the age range of >60 y. Studies of participants diagnosed with compromised metabolic or genetic health issues were excluded from review, e.g., individuals with diabetes, cardiovascular conditions, cancer cachexia, arthritis osteoporosis or any distinct chronic illness. This decision was taken because such conditions may impact post-prandial rates of MPS. Likewise, studies that included participants on any medications (e.g., diabetes medications), which may produce hypo- or hyper anabolic stimuli, were excluded.

#### Types of Interventions

This systematic review was limited to interventions that administered protein in a single oral bolus, and compared post-prandial blood leucine concentration profiles and rates of MPS between two or more protein-based interventions. Dietary protein could be provided in supplement form (isolated whey, micellar casein, soy, wheat, collagen) or in food form (milk and beef), but interventions had to be matched for protein dose. Studies that included an exercise (resistance, aerobic, or concurrent) stimulus also were included.

#### Types of Outcome Measurements

The primary outcome measurement from eligible studies was a qualitative appraisal of the leucine trigger hypothesis, i.e., sufficient evidence that blood leucine concentration profiles correspond with post-prandial rates of MPS, or insufficient evidence that blood leucine concentration profiles correspond with post-prandial rates of MPS. This approach was based on the statistical outcomes for measurements of post-prandial blood leucine concentrations and MPS when compared between protein conditions within the same study. Hence, if “protein condition A” resulted in both a greater blood leucine response and MPS response than “protein condition B,” the study was classified as “yes,” e.g., providing support for the leucine trigger hypothesis. In contrast, the study was classified as “no” if “protein condition A” resulted in a greater blood leucine response than “protein condition B,” but the MPS response was not statistically different between conditions or a greater MPS response was observed in “protein condition B.” Blood leucine concentration profiles were determined by measurements of plasma leucine concentrations, expressed as peak values during the post-prandial period or as area under the curve (AUC) to represent the “overall” leucine response over the entire post-prandial period. Post-prandial rates of MPS were measured over the same time period, thus enabling us to determine the correspondence between blood leucine concentration profiles and post-prandial rates of MPS in response to an ingested protein source. All included studies assessed MPS by calculating the fractional synthesis rate (FSR) of muscle proteins using the gold standard precursor-product approach. Included studies assessed either mixed-muscle or myofibrillar protein synthesis rates.

### Data Collection and Analysis

#### Selection of Studies

The eligibility of study titles and abstracts generated by the literature search was performed by two reviewers (G Zaromskyte and T Ioannidis). Studies that matched the criteria were reserved and full texts obtained for further screening. Full texts were subsequently screened by two independent reviewers (K Prokopidis and O Witard) based on the eligibility criteria detailed above. Any disagreements between reviewers were resolved by consensus. All records generated by the literature search on PubMed, Scopus, and Ovid MEDLINE and EMBASE were managed using the reference management software EndNote (Thomson Reuters, version X7).

#### Data Extraction and Management

Two reviewers (G Zaromskyte and K Prokopidis) extracted all data (i.e., participant characteristics, blood leucine concentration profiles, post-prandial rates of MPS) from included studies using a customized table. Data were organized based on study participant age and whether post-prandial rates of MPS were measured in the rested or post-exercise state. Categories of data extracted included descriptive information on participant characteristics (age, sex, and physical activity status), study design/intervention (i.e., details of protein sources), methodological details regarding measurement of MPS (mixed or myofibrillar muscle protein fraction, tracer incorporation period), and details of data outcomes (i.e., qualitative appraisal supporting or refuting the leucine trigger hypothesis (yes or no) and main findings.

#### Method of Data Synthesis

Data from included studies were synthesized qualitatively as a quantitative analysis was not appropriate given the heterogenous nature of between laboratory assessments of MPS (21). As part of the data extraction process, reviewers were required to synthesize datasets for each study to determine whether there was sufficient evidence to support the leucine trigger hypothesis. Sufficient evidence of the leucine trigger included a data set whereby a greater blood leucine concentration profile corresponded with higher rates of MPS during the post-prandial period. Following extraction, data were synthesized based on the age of participant studied and whether post-prandial rates of MPS were measured under resting or post-exercise conditions.

## Results

### Literature Search

**Figure 1:**
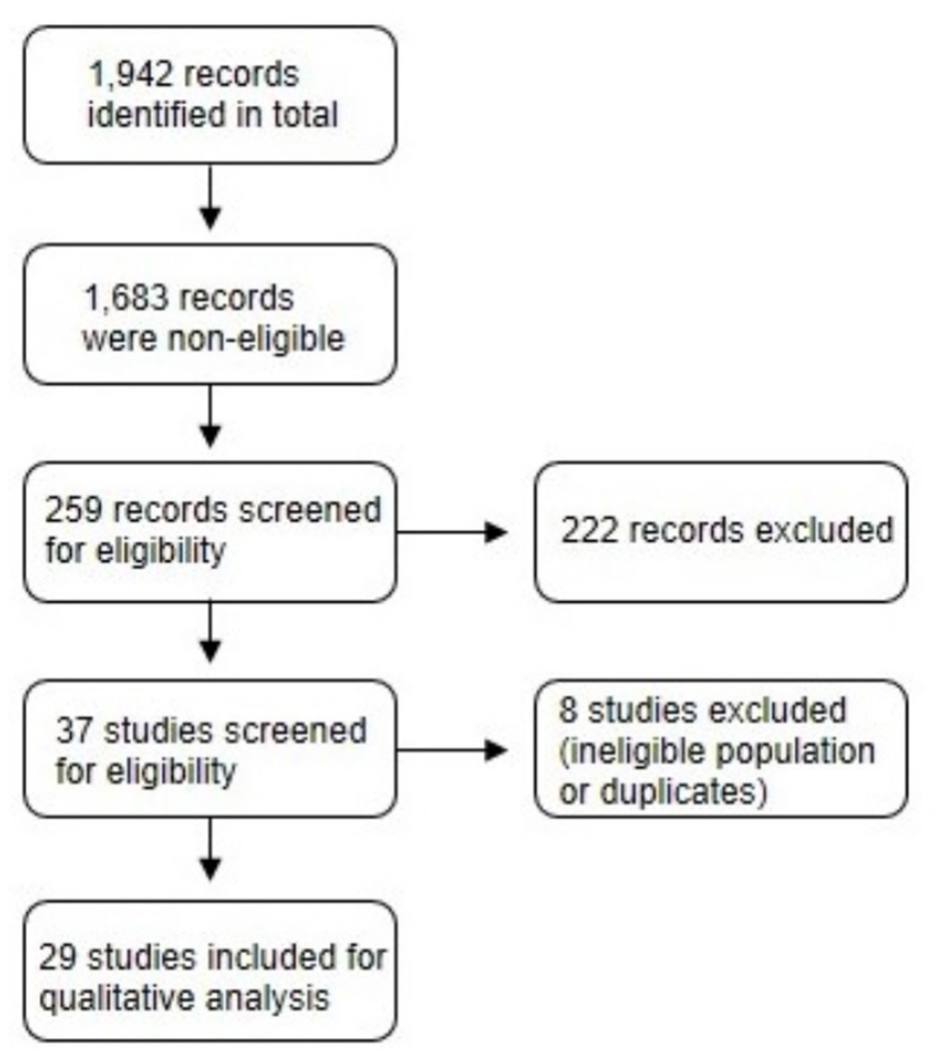
A flow diagram of the screening process for selecting eligible studies.

Figure 1 displays the screening process for selecting eligible studies. A total of 1,942 records were produced by the literature search. Of this total, 1,683 records were removed because they were either conducted in animals or in human subjects with a pre-existing health condition. A total of 37 studies were screened and 8 were excluded due to ineligible population characteristics or duplicates. A final sample of 29 studies were included for qualitative analysis.

### Included Studies

Tables 1–4 detail all studies included in the systematic review. Among the selected studies, a large heterogeneity across studies was identified in terms of participant characteristics, type of ingested protein, and exercise modality (resistance/aerobic/concurrent exercise). Tables 1, 2 display the summary of findings from studies that measured blood leucine concentration profiles and post-prandial rates of MPS in older and young adults at rest, whereas Tables 3, 4 display the summary of findings from studies that measured blood leucine concentration profiles and post-prandial rates of MPS in older and young adults following exercise.

**Table 1:** Summary of findings from studies that measured blood leucine concentration profiles and post-prandial rates of muscle protein synthesis at rest in older adults.

**References**	**Participants**	**Study design/Intervention**	**Muscle fraction for post-prandial MPS measurement**	**Evidence supporting “leucine trigger” hypothesis**	**Blood leucine concentration profile**	**Post-prandial rates of MPS profile**
Fuchs et al. (22)	Healthy, untrained males (71 ± 1 yr)	Double-blinded, Parallel RCT 6 g BCAA (*n* = 15) 6 g BCKA (*n* = 15) 30 g milk protein (*n* = 15)	Myofibrillar 0–5 h	No	Peak plasma leucine concentrations: BCAA (45–60 min) > Milk (75–90 min) > BCKA (45–60 min). Overall plasma leucine concentrations: BCAA > Milk > BCKA.	Milk (0.022 ± 0.002%/h) = BCAA (0.022 ± 0.002%/h) = BCKA (0.021 ± 0.001%/h) at 0–2 h from BL. Milk (0.039 ± 0.004 %/h) > BCAA (0.024 ± 0.005 %/h) = BCKA (0.024 ± 0.005%/h) at 2–5 h.
Devries et al. (23)	Healthy, untrained females (69 ± 1 yr)	Single-blinded, parallel RCT 15 g milk (4.2 g LEU) (*n* = 11) 15 g milk + soy (1.3 g LEU) (*n* = 11)	Myofibrillar 0–4 h	Yes	Peak and overall plasma leucine concentrations: Milk > milk + soy.	Milk>milk + soy; +53% from BL vs. +13% from BLCorrelation between peak plasma leucine concentrations and MPS:(*r* = 0.57, *P* = 0.01)
Reitelseder et al. (24)	Healthy, moderately active males (69 ± 1 yr)	Single-blinded, RCT 0.45 g/kg LBM WH (*n* = 10) 0.45 g/kg LBM CAS (*n* = 9)	Myofibrillar 0–3 h	No	Overall plasma leucine concentrations: WH > CAS at 15–90 min.	CAS (0.045 ± 0.003%/h) = > WH (0.043 ± 0.004%/h)
Kouw et al. (25)	Healthy, untrained males (72 ± 1 yr)	Double-blinded, parallel RCT PLA (*n* = 12) PRO20 [20 g casein (*n* = 12)] PRO20 + LEU [20 g casein and 1.5 g LEU (*n* = 12)] PRO40 [40 g casein (*n* = 12)]	Myofibrillar 0–7.5 h	Yes	Peak plasma leucine concentrations: PRO20 + LEU (396 ± 20 μM) > PRO40 (316 ± 19 μM) > PRO20 (269 ± 10 μM) at 30–180 min. PRO40 > PRO20 + LEU at 180–480 min.	L-[ring-2H5]-phenylalanine) PRO40 (0.044 ± 0.003%/h) > PRO20 + LEU (0.039 ± 0.002%/h) > PRO20 (0.037 ± 0.003%/h) > PLA (0.033 ± 0.002%/h). L-[1-13C]-leucine PRO40: (0.058 ± 0.003%/h) > PRO20 + LEU (0.056 ± 0.002%/h) > PRO20 (0.046 ± 0.004%/h) = PLA (0.047% ± 0.004%/h).
Gorissen et al. (26)	Healthy, untrained males (71 ± 1 yr)	Double-blinded, parallel RCT 35 g wheat (*n* = 12) (2.5 g LEU) 35 g WPH (*n* = 12) (2.5 LEU) 35 g micellar casein (*n* = 12) (3.2 g LEU) 35 g whey (*n* = 12) (4.4 g LEU) 60 g WPH (*n* = 12) (4.4 g LEU)	Myofibrillar 0–4 h	No	Peak plasma leucine concentrations: 35g whey (580 ± 18 μM) > 60g wheat (378 ± 10 μM).	35 g micellar casein > Whey > Wheat at 0–4h60 g WPH > 35 g whey at 2–4 h. Micellar casein (0.050% ± 0.005%/h) > 60 g WPH (0.049% ± 0.007%/h) > 35 g WPH (0.032% ± 0.004%/h).
Churchward-Venne et al. (27)	Healthy, untrained males (71 ± 1 yr)	Parallel RCT*n* = 32 25 g bovine milk serum casein 25 g casein protein	Myofibrillar 0–5 h	No	Overall plasma leucine concentrations: Casein > Bovine milk serum casein at 30–180 min.	Bovine milk serum casein = casein at 0–2 h.(0.038 ± 0.005 vs. 0.031 ± 0.007%/h). Casein > Bovine milk serum casein at 2–5 h.(0.067 ± 0.005 vs. 0.052 ± 0.004 %/h).
Mitchell et al. (28)	Healthy, middle-aged sedentary to recreationally active males WPC (52.6 ± 3.9 yr) MPC (52.1 ± 6.4 yr)	Double-blinded RCT 20 g WPC (2.3 g LEU; *n* = 8) 20 g MPC (2.1 g LEU; *n* = 8)	Myofibrillar 0–3.5 h	No	Plasma leucine concentrations: WPC > MPC at 45 and 75 min	WPC (0.021 ± 0.018 %/h) > MPC (0.019 ± 0.009 %/h) at 0–210 min MPC (0.057 ± 0.018 %/h) > WPC (0.052 ± 0.024 %/h) at 0–90 min
Wall et al. (29)	Healthy, untrained males (74 ± 1 yr)	Parallel RCT CAS + LEU [20 g casein (*n* = 12)] CAS [20 g casein and 2.5 g LEU (*n* = 12)]	Mixed 0–6 h	Yes	Overall plasma leucine concentrations: CAS + LEU > CAS at 30–180 min.	CAS + LEU (0.0078 ± 0.001 %/h) > CAS (0046 ± 0.001 %/h) at 0–2 h. CAS + LEU (0.023 ± 0.002 %/h) > CAS (0.019 ± 0.001 %/h) at 2–6 h. CAS + LEU (0.049 ± 0.003 %/h) > CAS (0.040 ± 0.003 %/h) at 0–6 h.
Pennings et al. (30)	Healthy, untrained males (74 ± 1 yr)	Parallel RCT 20 g whey (*n* = 16) 20 g casein (*n* = 16) 20 g casein hydrolysate (*n* = 16)	Mixed 0–6 h	Yes	Peak plasma leucine concentrations: Whey (526 ± 21 μM) > casein hydrolysate (381 ± 14 μM) > casein (282 ± 13 μM).	Whey (0.15 ± 0.02%/h) > Casein hydrolysate (0.10 ± 0.01%/h) > Casein (0.08 ± 0.01%/h). Strong positive (*r* = 0.66) correlation between plasma leucine concentrations and mixed MPS.
Koopman et al. (31)	Healthy, untrained males (64 ± 1 yr)	Crossover, Double-blinded trial*n* = 10CAS (35 g intact casein) or CASH (35 g hydrolyzed casein)	Mixed 0–6 h	Yes	Overall plasma leucine concentrations: CASH: 42.7 ± 2.3 > CAS: 32.6 ± 1.8 μmol·6 h/kg (AUC).	CASH (0.068 ± 0.006 %/h) > CAS (0.054 ± 0.004 %/h).

*AUC, area under curve; BCAA, branched-chain amino acids; BCKA, branched-chain keto acids; BL, baseline; LEU, leucine; MPC, milk protein concentrate; MPS, muscle protein synthesis; PLA, placebo; RCT, randomized controlled trial; WPC, whey protein concentrate; WPH, wheat protein hydrolysate*.

*Values are presented as means ± SE*.

**Table 2:** Summary of findings from studies that measured blood leucine concentration profiles and post-prandial rates of muscle protein synthesis at rest in younger adults.

**References**	**Participants**	**Study Design/Intervention**	**Muscle fraction for MPS measurement**	**Evidence supporting “leucine trigger” hypothesis**	**Blood leucine concentration profile**	**Post-prandial rates of MPS profile**
Pinckaers et al. (32)	Healthy, recreationally active males (23 ± 3 yr)	Double-blind, Parallel RCT 30 g milk protein (*n* = 12) 30 g wheat + milk protein (*n* = 12) 30 g wheat (*n* = 12)	Myofibrillar 0–5h	No	Peak plasma leucine concentrations: Milk (353 ± 45 μM) > wheat + milk (301 ± 44 μM) > wheat (280 ± 37 μM). Overall plasma leucine concentrations (AUC): Milk (36 ± 7 mmol·300 min/L) > wheat + milk (25 ± 9 mmol·300 min/L) > wheat (22 ± 3 mmol·300 min/L).	Wheat + milk (0.067 ± 0.032 %/h) > milk (0.059 ± 0.024 %/h) > wheat (0.053 ± 0.025 %/h) at 0–2 h. Wheat (0.058 ± 0.013 %/h) > Wheat + milk (0.054 ± 0.036 %/h) > milk (0.049 ± 0.017 %/h) at 2–5 h. Wheat + milk (0.059 ± 0.025 %/h) > wheat (0.056 ± 0.012 %/h) > milk (0.053 ± 0.013 %/h) at 0–5 h.
Luiking et al. (33)	Healthy, untrained males and females (23 ± 1 yr)	Single-blinded, RCT 0.21 g/kg/bw casein (*n* = 12) 0.21g/kg/bw soy (*n* = 10)	Mixed 0–4h	Yes	Overall plasma leucine concentrations: Soy (128 ± 13 μM) > casein (95 ± 7 μM) at 0 h. Soy (117 ± 9 μM) < casein (121 ± 5 μM) at 4 h.	Soy > casein.

*AUC, area under curve; MPS, muscle protein synthesis; RCT, randomized controlled trial*.

*Values are presented as means ± SE*.

**Table 3:** Summary of findings from studies that measured blood leucine concentration profiles and post-prandial rates of muscle protein synthesis following exercise in older adults.

**References**	**Participants**	**Study Design/Intervention**	**Muscle fraction for MPS measurement**	**Evidence supporting “leucine trigger” hypothesis**	**Blood leucine concentration profile**	**Post-prandial rates of MPS profile**
Oikawa et al. (34)	Healthy, untrained females (69 ± 3 yr)	Double-blinded, parallel RCT30 g whey (4.3 g LEU) (*n* = 11) 30 g collagen (0.9 g LEU) (*n* = 11) Unilateral leg extension, 4 × 8–10 reps @ 60% 1RM	Mixed 0–4 h	Yes	Overall plasma leucine concentrations (AUC): Whey (103,800 ± 17,000 μmol min/L) > collagen (43,600 ± 10,100 μmol ·min/L) Peak plasma leucine concentrations: Whey (645 ± 206 μM) > collagen (223 ± 117 μM)	Whey>Collagen Whey: 0.017 ± 0.008%/h (rest); 0.032 ± 0.012%/h from BL Collagen: 0.009 ± 0.014%/h (rest); 0.012 ± 0.013%/h from BL
Hamarsland et al. (35)	Healthy, trained males and females (74 ± 3.5 yr)	Double-blinded, partial crossover, RCT 20 g milk (2 g LEU) (*n* = 10) 20 g native whey (2.7 g LEU) or 20 g WPC (2.2 g LEU) (*n* = 11) 4 × 8 reps on leg press and leg extension @ 50–80% 1RM	Mixed 0–5 h	Yes	Overall plasma leucine concentrations: Native whey 45% > WPC (AUC). Native whey 130% > milk (AUC). WPC 60% > milk (AUC).	Native whey > WPC > milk.
Holwerda et al. (36)	Healthy, untrained males (67 ± 1 yr)	Double-blinded, RCT 15 g milk (milk) (*n* = 12) 15 g milk + 1.5g LEU (milk+LEU) (*n* = 12) 5 × 10 reps on horizontal leg press2 × 10 reps on latissimus dorsi pulldown2 × 10 reps on chest press5 × 10 reps on leg extension@ 50–80% 1RM	Mixed 0–6 h	Yes	Overall plasma leucine concentrations: Milk + LEU > milk at 0–2 h. Peak plasma leucine concentrations: Milk + LEU (407 ± 23 μM) > milk (234 ± 16 μM), at 30 min	(L-[*ring*-2H5]phenylalanine) Milk + LEU (0.0575 ± 0.0032%/h) > milk (0.0495 ± 0.0021%/h) (L-[1-13C]leucine) Milk + LEU (0.0710 ± 0.0048 %/h) > milk (0.0598 ± 0.0030 %/h)
Devries et al. (23)	Healthy, untrained females (69 ± 1 yr)	Single-blinded, parallel RCT 15 g milk (4.2 g LEU) (*n* = 11) 15 g milk + soy (1.3 g LEU) (*n* = 11)Unilateral leg extension exercise(2 sets @ 50% 1RM; 2 sets @ 60% 1RM)	Myofibrillar 0–4 h	Yes	Peak and overall plasma leucine concentrations: Milk > milk + soy.	Milk +87% > milk + soy + 30% from BLCorrelation between peak plasma leucine concentrations and MPS:(*r* = 056, *P* = 0.01)
Devries et al. (37)	Healthy, untrained females (69 ± 1 yr)	Single-blinded, parallel RCT 24.9 g WPI (3 g LEU) (*n* = 11) 10.0 g milk (3 g LEU) (*n* = 11)Unilateral leg extension exercise(4 sets @ 50–60% 1RM)	Myofibrillar 0–4 h	Yes	Overall plasma leucine concentrations: Milk > WPI at 0–45 min. Milk < WPI at 120–240 min. Peak plasma leucine concentrations: Milk>WPI.	WPI > milk, +63% from BL vs. +58% from BL (rest) WPI = milk, +9% from BL for WPI and milk (post-exercise)
Reitelseder et al. (24)	Healthy, moderately active males (69 ± 1 yr)	Single-blinded, RCT 0.45 g/kg LBM WH (*n* = 10) 0.45 g/kg LBM CAS (*n* = 9)Unilateral leg extensions(10 sets × 8 reps @ 70% 1RM)	Myofibrillar 0–3 h	No	Overall plasma leucine concentrations: WH > CAS at 15–90 min.	CAS (0.043 ± 0.004%/h) = WH (0.041 ± 0.004%/h)
Wilkinson et al. (38)	Healthy, untrained females (65 ± 1 yr)	Parallel RCT LEAA_1.5 (0.6 g LEU; 1.5 g EAA) (*n* = 8) LEAA_6 (2.4 g LEU; 6 g EAA) (*n* = 8) Whey [40 g whey (4 g LEU) (*n* = 8)]Unilateral knee extensions(6 sets × 8 reps @ 75% 1RM)	Myofibrillar 0–7 h	No	Overall plasma leucine concentrations: Whey > LEAA_6 > LEAA_1.5 at 60–240 min.	LEAA_6=LEAA_1.5=Whey (rest). Whey > LEAA_6 > LEAA_1.5 (post-exercise).
Borack et al. (39)	Healthy, recreationally active males WPI (69.3 ± 2.1 yr) PB (62.2 ± 1.5 yr)	Double-blinded, RCT 30.4 g WHPI (3.26 g LEU; *n* = 10) 30.5 g PB (2.8 g LEU; *n* = 9)Leg extensions 8 × 10 reps (sets 4–8 @70% 1RM)	Mixed 0–4 h	Yes	Overall plasma leucine concentrations (AUC): WPI = PB	WPI (0.09 ± 0.01%) = PB (0.09 ± 0.01%)
Bukhari et al. (40)	Healthy, untrained females (66 ± 3 yr)	Parallel RCT LEAA [3 g EAA (1.2 g LEU)] (*n* = 8) WP [20 g whey (2 g LEU)] (*n* = 8)Unilateral leg extension, 6 × 8 reps @ 75% 1RM	Myofibrillar 0–4 h	Yes	Overall plasma leucine concentrations: WP > LEAA at 60–220 min.	WP (0.016 ± 0.003 %/h) = LEAA (0.018 ± 0.004 %/h) (rest) WP (0.029 ± 0.007 %/h) > LEAA (0.014 ± 0.010 %/h)(post-exercise)
Burd et al. (16)	Healthy, active males (72 ± 1 yr)	Parallel RCT 20 g micellar casein (*n* = 7) 20 g whey (*n* = 7)Unilateral leg extension, 3 sets@ 10 RM	Myofibrillar 0–4 h	Yes	Overall plasma leucine concentrations (mean): Whey (193 ± 17 μM) > micellar casein (175 ± 17 μM). Peak plasma leucine concentrations: Whey (296 ± 20 μM) > micellar casein (202 ± 21 μM) at 60 min.	Whey > micellar casein
Dideriksen et al. (41)	Healthy, moderately active males and females (68 ± 1 yr)	Parallel RCT*n* = 24 50 g whey (11.8 g LEU) 46.5 g caseinate (8.8 g LEU) 5 × 8 on leg press and knee extensions @ 80% 1RM	Myofibrillar 0–6.5 h	No	Peak plasma leucine concentrations: Whey (490 ± 32 μmol/L) > Caseinate (282 ± 17 μmol/L)	Whey (0.09 ± 0.005%/h) = Caseinate (0.09 ± 0.003%/h)

*AUC, area under curve; BL, baseline; CAS, caseinate protein; EAA_HL, essential amino acids with high leucine; EAA_LL, essential amino acids with low leucine; LEAA, leucine-enriched essential amino acids; LEU, leucine; MPS, muscle protein synthesis; PB, soy-dairy protein blend; RCT, randomized controlled trial; RM, repetition maximum; WH, whey hydrolysate; WPC, whey protein concentrate; WPI, whey protein isolate*.

*Values are presented as means ± SE*.

**Table 4:** Summary of findings from studies that measured blood leucine concentration profiles and post-prandial rates of muscle protein synthesis following exercise in young adults.

**References**	**Participants**	**Study Design/Intervention**	**Muscle fraction for MPS measurement**	**Evidence supporting “leucine trigger”hypothesis**	**Blood leucine concentration profile**	**Post-prandial rates of MPS profile**
Churchward-Venne et al. (42)	Healthy, recreationally active males (23 ± 0.4 yr)	Double-blinded, parallel RCT 20 g whey (2.6 g LEU) (*n* = 12) 20 g soy (1.44 g LEU) (*n* = 12) 20 g soy + LEU (2.6 g LEU) (*n* = 12) 4 × 8 reps on leg press and leg extension machine (80% 1RM) and 30 min static cycling (60% Wmax)	Mixed 0–6 h	No	Overall plasma leucine concentrations: Whey > Soy + LEU > Soy (AUC) Peak plasma leucine concentrations: Soy + LEU (328 ± 14 μM; +165% from BL) > Whey group (322 ± 10 μM; +152% from BL) > Soy (216 ± 6 μM; +75% from BL, at 30–180 min.	Whey (0.054 ± 0.002%/h) = Soy (0.053 ± 0.004%/h) = Soy +Leu (0.056 ± 0.004%/h)
Churchward-Venne et al. (43)	Healthy, recreationally active males (23 ± 0.3 yr)	Double-blinded, parallel RCT 20 g milk (1.7 g LEU) (*n* = 12) 20 g whey (2.6 g LEU) (*n* = 12) 20 g micellar casein (2 g LEU) (*n* = 12) 4 × 8 reps on leg press (80% 1RM) and 30 min cycling (60% VO2max)	Myofibrillar 0–6 h	No	Peak plasma leucine concentrations: whey (322 ± 10 μmol/L) > micellar casein (245 ± 5 μmol/L) > milk (242 ± 8 μmol/L)	Milk (0.059 ± 0.003%/h) = Casein (0.059 ± 0.005%h) > Whey (0.054 ± 0.002%/h)
Chan et al. (44)	Healthy, untrained males (22.5 ± 3.0 yr)	Parallel RCT 25 g MPC (2.6 g LEU) (*n* = 10) 25 g mMPC (2.6 g LEU) (*n* = 10) 25 g CAS (2.35 g LEU) (*n* = 10)3 sets on leg press (80% 1RM)and 3 sets on leg extensions (80% 1RM)	Myofibrillar 0–4 h	Yes	Overall plasma leucine concentrations: mMPC > CAS by 58% from BL mMPC > MPC by 54% from BL, both at 30–90 min.	CAS by (140.6 ± 52.4%) > mMPC by (137.8 ± 72.1%) > MPC by (82.6 ± 64.8%) from BL
Trommelen et al. (45)	Healthy, recreationally active males (24 ± 1 yr)	Double-blinded, RCT PRO (30 g casein) (*n* = 12) PRO + LEU (30 g casein protein + 2 g LEU) (*n* = 12) 6 × 10 reps on horizontal leg press and leg extension	Mixed 0–7.5 h	No	Overall plasma leucine concentrations: PRO + LEU > PRO at 30–300 min.	(L-[*ring*-^2^H_5_]phenylalanine)PRO + LEU (0.055 ± 0.004%/h) = PRO (0.055 ± 0.002 %/h) (L-[1-13_C_]leucine)PRO + LEU (0.083 ± 0.006%/h) > PRO (0.073 ± 0.004 %/h)
Burd et al. (19)	Healthy, recreationally active males (22 ± 1 yr)	Crossover RCT*n* = 12 30 g skimmed milk protein (2.7 g LEU) or 30 g minced beef protein (2.5 g LEU) 4 × 8-10 reps on leg press and extension	Mixed 0–5 h	No	Overall plasma leucine concentrations: Milk > beef at 30 min. Beef > milk at 60–120 min. Peak plasma leucine concentrations: Beef (277 ± 12 μM @ 115 min) > Milk (231 ± 11 μM @ 135 min).	Milk by (128% ± 23%) > Beef by (91% ± 15%) at 0–2 h from BL. Milk (0.071 ± 0.005%/h) > Beef (0.057 ± 0.006 %/h) at 0–5 h.
Reidy et al. (46)	Healthy, recreationally active males and females (WPI; 23.1 ± 1.0 yr; PB; 25.1 ± 1.2 yr)	Double-blinded, RCT*n* = 1919 g PB (1.8 g LEU; 8.7 g EAA) 18 g WPI (1.9 g LEU; 8.9 g EAA) >8 × 10 on leg extension (55–70% 1RM)	Mixed 0–4 h	No	Overall plasma leucine concentrations: WPI > PB at 20–120 min.	PB (0.088 ± 0.007%/h) = WPI (0.078 ± 0.009%/h) at 0–2 h; similar increase from BL PB (0.087 ± 0.003%/h) > WPI (0.074 ± 0.010%/h)at 2–4 h.
Tang et al. (15)	Healthy, resistance-trained males (22.8 ± 3.9 yr)	Parallel RCT 21.4 g whey (10 g EAA; 2.3 g LEU) (*n* = 6) 21.9 g casein (10 g EAA; 1.8 g LEU) (*n* = 6) 22.2 g soy (10 g EAA; 1.8 g LEU) (*n* = 6)Unilateral leg press and leg extension4 × 10–12 RM	Mixed 0–3 h	Yes	Overall plasma leucine concentrations: Whey > soy by 74% from BL Whey > casein by 200% from BL	Whey (0.091 ± 0.015 %/h) > soy (0.078 ± 0.014 %/h) > casein (0.047 ± 0.008%/h) (rest) Whey > soy by 31% (post-exercise) from BL Whey > casein by 122% (post-exercise) from BL
Wilkinson et al. (47)	Healthy, resistance-trained males (23.1 ± 0.3 yr)	Single-blinded, RCT*n* = 818 g protein; calorie-matched milk or soy beverages4 × 10 reps on leg press, hamstring curl and knee extension @ 80% 1RM	Mixed 0–3 h	No	Overall whole-blood total amino acid concentrations: Soy ~ Milk at 60–180 min Peak whole-blood total amino acid concentrations: Soy (25 μmol/L) > Milk (14 μmol/L) at 30 min Soy ↓ 9 μmol/min; Milk ↓ 0.8 μmol/min at 30–60 min Muscle leucine concentrations: Milk (0.69 ± 0.06 mmol/kg) > Soy (0.59 ± 0.04 mmol/kg) at 60 min Milk (0.55 ± 0.03 mmol/kg) > Soy (0.54 ± 0.04 mmol/kg) at 120 min Milk (0.54 ± 0.04 mmol/kg) > Soy (0.44 ± 0.02 mmol/kg) at 180 min	Milk (0.10 ± 0.01 %/h) > Soy (0.07 ± 0.01 %/h)

*AUC, area under curve; BL, baseline; CAS, calcium caseinate; EAA, essential amino acids; LEU, leucine; mMPC, modified milk protein concentrate; MPC, milk protein concentrate; MPS, muscle protein synthesis; PB, protein blend; RCT, randomized controlled trial; VO_2_max, maximal oxygen uptake; Wmax, maximal power output in watts; WPI, whey protein isolate*.

*Values are presented as means ± SE*.

### Participants

All participants across studies were healthy, as defined by the absence of metabolic conditions, no prescription medication, no smoking or excessive alcohol use and a BMI <30. Overall, 18 studies recruited males, five studies recruited females, and six studies recruited both males and females. At rest, 10 studies recruited older adults (1 middle-aged; 8 in males only, 2 females) and two studies recruited younger adults (1 in males only; 1 in males and females combined). Of the eight studies in young adults, six recruited exercise trained individuals. Following exercise, 11 studies recruited older adults (5 females; 4 males; 2 females and males) and eight studies recruited young adults (6 males; 2 males and females). Two studies (23, 24) were included in both Tables 1, 3 since the measurement of MPS (and blood leucine concentrations) was conducted under both rested and exercised conditions in older adults.

### Details of Anabolic Interventions

Of the 28 studies, 18 measured post-prandial rates of MPS in response to ingested protein plus exercise, 11 studies measured post-prandial rates of MPS in a rested state, while two measured post-prandial rates of MPS in both resting and post-exercise states. Only interventions that included the oral administration of protein (physiologically relevant) were included in the systematic review, as opposed to studies that administered an amino acid source intravenously (not relevant to leucine trigger hypothesis since a square wave in amino acid appearance is clamped without fluctuation of magnitude). The anabolic interventions were isolated proteins, including whey (16 study arms), casein (14 study arms), soy (4 study arms), wheat (2 study arms), and collagen (1 study arm), as well as protein-rich foods including milk (10 study arms), milk + soy (1 study arm), wheat + milk (1 study arm), and beef (1 study arm).

### Experimental Methodology and Quality Assessment

Of the 29 studies, 14 were double-blinded, four were single-blinded and 11 were unblinded. Moreover, two studies utilized a cross-over research design, whereas 16 studies were parallel in design with participants either in experimental or control groups. Similarly, physical activity prior to the experiment was monitored across studies, mostly for 2 d by requesting that participants refrain from exercise for this period. With regards to the measurement of MPS, 14 studies measured MPS at the mixed protein level and 15 studies measured MPS in the myofibrillar fraction. Muscle biopsies for measurement of MPS were obtained from the *vastus lateralis* in all studies. Finally, the incorporation period for assessment of MPS ranged from 0 to 3 h post feeding to 0–7.5 h post feeding.

### Data Synthesis

**Figure 2:**
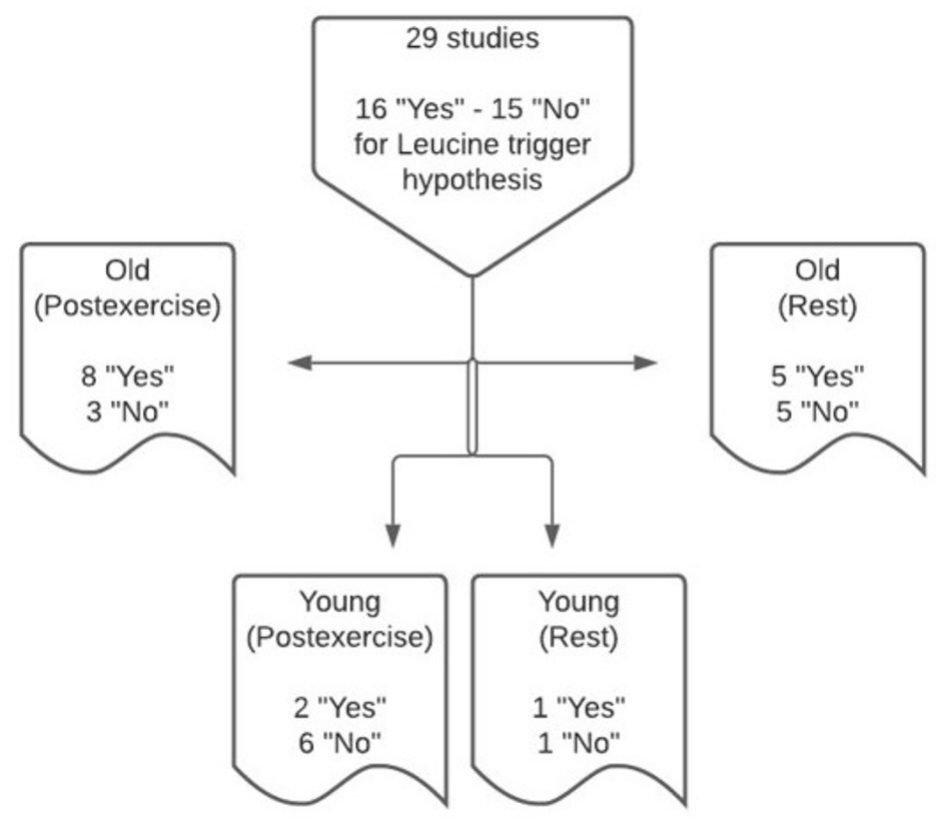
Synthesis of findings from 29 studies that support or refute the leucine trigger hypothesis to explain the post-prandial regulation of muscle protein synthesis in young and older adults at rest and following exercise, using 31 study arms overall.

The leucine trigger hypothesis was examined in 29 eligible studies, comprising 31 study arms, under resting and post-exercise conditions in young and older adults (Figure 2). Two studies (23, 24) conducted measurements of plasma leucine concentrations and post-prandial rates of MPS under both resting and post-exercise conditions (Tables 1, 3). Data from five studies in older adults at rest provide evidence to support the leucine trigger hypothesis for stimulating MPS, whereas five studies did not support the leucine trigger hypothesis. In the post-exercise state, eight studies of older adults support the hypothesis, whereas three studies (one in middle-aged adults) reported a greater MPS response when blood leucine concentrations were lower during the post-prandial period. In young adults at rest, data from one study support the leucine trigger hypothesis, whereas a disconnect between blood leucine concentration profiles and post-prandial rates of MPS also was observed in one study. In the post-exercise state, two studies of young adults supported the leucine trigger hypothesis, whereas six studies reported a greater MPS response when blood leucine concentrations were lower during the post-prandial period.

## Discussion

The primary aim of this systematic review was to evaluate the role of the leucine trigger hypothesis to explain post-prandial rates of MPS in response to protein ingestion in young and older adults.

Overall, this systematic review revealed that 16 study arms support the leucine trigger hypothesis to explain the post-prandial regulation of MPS, whereas 15 study arms refute the hypothesis that a more rapid rate of appearance (magnitude and/or duration) of leucine into the circulation is stimulatory for increasing post-prandial rates of MPS. Indeed, two study arms observed a more modest profile of blood leucine concentrations to correspond with greater rates of MPS. We have identified four key factors that contribute to the discrepant findings, namely (i) the dose of protein, (ii) exercise status, (iii) the type/source of ingested protein, and (iv) methodological considerations primarily related to the measurement of MPS.

### Protein Dose

The equivocal findings regarding the application of the leucine trigger hypothesis to explain differential post-prandial rates of MPS in response to ingested protein may be related, at least in part, to the age of studied participants. Perhaps surprisingly, our findings indicate that the strength of evidence supporting the leucine trigger hypothesis is greater in older vs. young adults. In this regard, only three study arms in young adults provide evidence supporting the leucine trigger hypothesis (15, 33, 44), while seven studies refute the hypothesis (19, 32, 42, 43, 45–47). In contrast, the preponderance of evidence in older adults supports the leucine trigger hypothesis, with 13 studies reporting a greater post-prandial leucinemia following protein ingestion to correspond with an increased stimulation of MPS, and only seven studies reported a disconnect between the blood leucine concentration profile and post-prandial rates of MPS. As such, this observation may have age-specific implications for optimizing protein-based nutrition recommendations for the maximal stimulation of MPS in young and older adults.

The phenomenon of muscle anabolic resistance describes the impaired stimulation of MPS in response to key anabolic stimuli (i.e., muscle loading and/or amino acid/protein provision) and is generally accepted to be a fundamental mechanism underpinning the age-related decline in skeletal muscle mass (21, 48). However, recent evidence highlights comparable post-prandial rates of MPS between young and older adults when the dose of ingested protein, and constituent leucine profile, exceeds a certain (leucine) “threshold” in older adults (21, 27). In this systematic review, a 20 g protein dose was typically administered in studies of older adults to mimic the protein content of a typical meal. Assuming a constituent amino acid profile of ~10% leucine, the total leucine content of ingested protein in these studies was equivalent to ~2 g of leucine that is below the 3 g leucine threshold proposed for the maximal stimulation of MPS in older adults (49). Hence, in the context of a meal-like dose of protein, our data support the notion that the amplitude of peak post-prandial leucinemia serves as key factor in regulating post-prandial rates of MPS in older adults. In contrast, the regulatory role of blood leucine availability in stimulating MPS becomes less apparent if the protein dose and leucine content is sufficient to stimulate a maximal post-prandial response of MPS (50, 51), as was the case in most studies of young adults included in this systematic review. By virtue of this age-related anabolic resistance phenomenon, alongside the inevitable decline in appetite and oral health associated with advancing age (52, 53), the administration of an “optimal” protein dose for maximal stimulation of MPS is more challenging in older adults. Accordingly, we present evidence that the leucine trigger hypothesis appears to confer greater application in explaining differences in post-prandial rates of MPS in older vs. young adults.

### Rest Vs. Exercise

The leucine trigger hypothesis was originally conceived, at least in humans, to explain divergent post-prandial rates of MPS in response to ingesting different isolated protein sources (i.e., whey, micellar casein and soy fractions) following exercise in healthy, trained, young men who engaged in whole-body resistance training at least 2 times per week (15, 54). Thereafter, this hypothesis has been extrapolated to encompass the post-prandial regulation of MPS at rest and following exercise in both young and older adult cohorts of both trained and untrained status. When pooling data for young and older adults, the findings from this systematic review indicate that the strength of evidence supporting the leucine trigger hypothesis is similar under post-exercise conditions (10/19 or 53% of studies support the hypothesis; Figure 2) and resting conditions (6/12 or 50% of studies support the hypothesis). However, when stratified by age, support for the hypothesis is stronger in older (8/11 or 73% of studies support the hypothesis) vs. young (2/8 or 25% of studies support the hypothesis) adults when assessed under post-exercise conditions. Taken together, these data suggest an interaction exists between age and exercise status with regards to supporting the leucine trigger hypothesis as an explanation for the regulation of MPS.

The notion that the leucine trigger applies only to exercise conditions has previously been challenged by two studies that manipulated the leucine content of a low dose of EAA (3 g) or whey protein (6.25 g) and measured post-prandial rates of MPS at rest and following exercise in young (55) and older (40) adults. In these studies, ingesting a leucine-enriched amino acid source elicited a robust increase in blood leucine concentrations and stimulated a similar response of MPS to the bolus ingestion of 20–25 g of whey protein at rest in both young and older adults (40, 55). Interestingly however, whereas ingesting the low dose leucine-rich EAA source stimulated similar post-exercise rates of MPS compared with 20 g of ingested whey protein in older adults (40), fortifying a low dose of whey protein with leucine failed to stimulate an equivalent post-exercise response of MPS to ingesting 25 g of whey protein in young adults, particularly during the later (3–5 h) exercise recovery period (55).

Intuitively, the authors reasoned that the capacity for a protein source to sustain an exercise mediated increase in MPS is not only dependent on extracellular leucine availability. Instead, an abundant supply of EAA (and potentially non-essential amino acids) also are required to provide additional substrate for the synthesis of new muscle proteins under conditions of higher “anabolic drive” stimulated by resistance exercise compared with feeding alone. The apparent disconnect between this thesis (55) and our observation that the leucine trigger hypothesis confers greater application during post-exercise conditions is difficult to reconcile, but may be explained by the range of different ingested protein sources included in this systematic review, particularly with regards to the potential interactive role of other nutrients (carbohydrate, lipids, fiber, and other bioactive constituents) within a food matrix in regulating post-prandial rates of MPS following the ingestion of protein-rich whole foods such as milk (19, 56), beef (19, 57), or pork (58). Unfortunately, a limited number of the studies included in this systematic review recruited previously trained individuals. Hence, the impact of training status on the role of the leucine trigger in modulating MPS warrants future investigation. This additional analysis is particularly interesting given the complex relationship between acute measurements of MPS and chronic changes in muscle mass (59). In this regard, the predictive value of acute measurements of MPS for chronic changes in muscle mass appears to be greater in trained vs. untrained individuals (60), suggesting that the leucine trigger hypothesis may be most relevant in trained individuals.

### Amino Acid/Protein Source

Burd et al. (20) recently proposed the idea that the leucine trigger hypothesis is more relevant within the context of ingesting isolated protein sources rather than protein-rich whole foods. This idea stems from the observation that ingesting protein-rich whole foods, such as skimmed milk or minced beef, are effective in stimulating a robust post-prandial increase in MPS, albeit in the absence of a rapid rise in leucinemia during post-exercise recovery in trained young men (19). This apparent disconnect between blood leucine concentration profiles and post-prandial rates of MPS in response to protein-rich foods contrasts with studies that administered isolated whey, soy and micellar casein fractions as fast, intermediate and slow proteins, respectively (15, 16). In these studies, the post-prandial response of MPS corresponded with the magnitude of leucinemia (as well as higher plasma EAA and BCAA concentrations), resulting in higher, intermediate and lower rates of MPS for whey, soy, and casein, respectively. The reason(s) behind these discrepant findings are yet to be fully elucidated, but may be related to the notion that other, non-protein, components within the whole food matrix are modulatory in regulating MPS.

The food matrix refers to the overall chemical dynamics of food, including how various food components are structured and interact (50). Consistent with this idea, a recent study demonstrated a greater post-prandial stimulation of MPS after ingesting whole eggs (egg white and yolk remained intact) than egg whites (egg yolk removed) during exercise recovery, despite a similar profile of blood leucine concentrations between egg conditions (51). Moreover, Elliot et al. (56) demonstrated that ingesting whole milk after exercise stimulated a greater amino acid uptake across the leg than fat-free milk when either matched for carbohydrate or energy content. Ultimately, this systematic review fails to provide additional insight into this theory given the limited number of studies that directly compare post-prandial rates of MPS in response to ingesting different whole protein foods. Hence, future mechanistic studies are warranted to elucidate the nutrient-nutrient interactions within the food matrix that may contribute to differential post-prandial rates of MPS following the ingestion of protein-rich food sources.

### Methodological Considerations

We cannot discount the possibility that methodological differences between studies, specifically in the measurement of MPS, may contribute to the mixed findings presented in this systematic review regarding the leucine trigger hypothesis. Such methodological considerations include, but may not be limited to, the duration of measurement for post-prandial rates of MPS, selection of muscle sub-fraction (i.e., mixed or myofibrillar) extracted for measurement of MPS, choice of isotopic tracer (i.e., ^13^C_6_ phenylalanine, 1- ^13^C leucine) and choice of precursor amino acid pool (plasma or intracellular) used to calculate fractional synthesis rates as the unit measurement for MPS (61). With regards to the duration over which post-prandial rates of MPS were measured, the tracer incorporation period ranged from 3 to 7.5 h within this systematic review, thus representing a wide range of measurement durations. Previous work demonstrates a transient post-prandial response of MPS that peaks ~3 h following protein ingestion (62). In theory, it follows that the leucine trigger may be more relevant within the early 0–3 h post-prandial period. Consistent with this notion, 10 of the 17 study arms that provide evidence to support the leucine trigger hypothesis measured MPS over a relatively short incorporation period, i.e., <4 h. Hence, in our hands, a link appears to exist between the duration of MPS assessment and support for the leucine trigger hypothesis. Moreover, previous studies have reported a differential response of MPS to exercise and/or nutritional stimuli dependent on whether mixed muscle or myofibrillar protein synthesis rates were measured (63). Whereas, the muscle intracellular amino acid pool arguably serves as a more accurate surrogate precursor for the calculation of MPS, for practical reasons (i.e., low tissue yield from biopsy) several studies, including some presented in this systematic review, used tracer enrichments in the blood amino acid pool as a more accessible precursor. Finally, discrepant findings have been reported for measurements of muscle protein metabolism within the same study based on choice of tracer infused (64). Taken together, it is feasible that these technical differences in methodology may contribute to the mixed findings regarding the regulatory role of the leucine trigger hypothesis.

## Conclusion

This systematic review is the first, to our knowledge, to qualitatively evaluate the leucine trigger hypothesis to explain the post-prandial regulation of MPS at rest and following exercise in young and older adults. In this systematic review, overall, 16 study arms (13 in older adults) provide evidence to support the hypothesis that the magnitude (amplitude and rate) of post-prandial increase in blood leucine concentrations, termed leucinemia, serves to regulate the magnitude of post-prandial MPS response to an ingested protein source. In contrast, 13 study arms refute the hypothesis. To conclude, these data underscore the merits of the leucine trigger hypothesis with greatest application in predicting the post-prandial response of MPS to ingested proteins in older adults. Moreover, and consistent with previous reports (20), we provide data to support the idea that the leucine trigger hypothesis is more relevant within the context of ingesting isolated protein sources rather than protein-rich whole foods. Moving forward, future studies should report more complete datasets that include basal measurements of MPS, thus allowing for the quantification of relative changes in MPS between conditions in follow-up systematic reviews and meta-analyses on this increasingly controversial topic of the leucine trigger hypothesis. Follow-up mechanistic studies also are warranted to understand the complex series of modulatory factors within a food matrix that regulate post-prandial rates of MPS.

## Data Availability Statement

The original contributions presented in the study are included in the article/supplementary material, further inquiries can be directed to the corresponding author.

## Author Contributions

GZ, TI, KT, and OW conceived and designed the research. GZ, TI, KP, and OW assisted with data analysis and result interpretation. GZ and KP prepared figures and tables. GZ, KP, and TI drafted manuscript. KP, KT, and OW revised manuscript. All authors approved final version of manuscript.

## Conflict of Interest

The authors declare that the research was conducted in the absence of any commercial or financial relationships that could be construed as a potential conflict of interest.
